# Scleredema in a Patient with AIDS-Related Lipodystrophy Syndrome

**DOI:** 10.1155/2013/943798

**Published:** 2013-01-10

**Authors:** Ralph Yachoui, Pamela Traisak, Shirish Jagga

**Affiliations:** Division of Rheumatology, Cooper Medical School of Rowan University, Suite 262, E and R Building, The Haddon Avenue Office, Camden, NJ 08103, USA

## Abstract

Scleredema is a form of cutaneous mucinosis caused by an increased accumulation of collagen and mucin in the dermis. It is characterized by diffused, nonpitting swelling and induration of the skin. Scleredema diabeticorum is one type of scleredema associated with diabetes mellitus. AIDS-related insulin resistance and lipodystrophy syndrome are a newly emerging entities in HIV-infected patients associated with severe metabolic disturbances and insulin resistance. The long-standing diabetes in these patients may contribute to the development of scleredema diabeticorum. Here, we report the rare occurrence of scleredema in an HIV-infected patient with AIDS-related lipodystrophy syndrome.

## 1. Introduction

Scleredema is a sclerosing skin condition, first described by Buschke in 1902, characterized by firm, nonpitting edema and indurated plaques that typically begin on the posterior neck and spread to the upper back, shoulders, and scalp. This condition may follow an infection or it can be associated with hyperinsulinemia and diabetes. However, some cases have neither association [1].

 AIDS-related insulin resistance and lipodystrophy syndrome is a newly emerging entity, which is observed in 30–80% of AIDS patients who are well controlled by highly active antiretroviral therapy (HAART). This syndrome is associated with severe metabolic disturbances, such as diabetes mellitus and dyslipidemia, which cause atherosclerotic cardiovascular disease [2]. In this paper, we describe the rare occurrence of scleredema in an HIV-infected patient with AIDS-related lipodystrophy syndrome.

## 2. Case

A 43-year-old man, HIV-seropositive since 1991 as a result of homosexual contact, presented with a three-month history of neck, shoulders, and upper back swelling with restricted mobility. He had been taking efavirenz, emtricitabine, and tenofovir for the last 5 years. He was treated of diabetes mellitus and dyslipidemia. He also had coronary artery disease status postpercutaneous coronary intervention.

**Figure 1:**
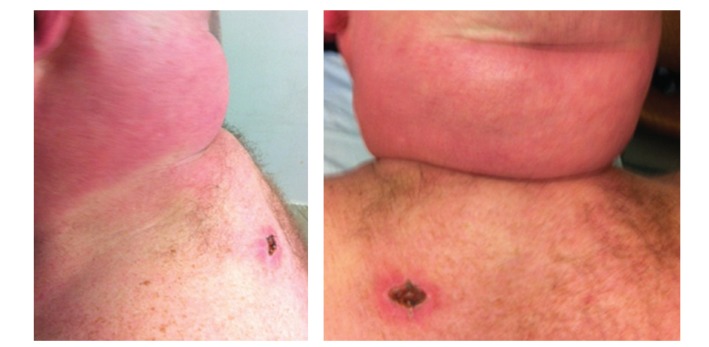
Nonpitting and thickened skin on upper back and nuchal area.

 On examination, he exhibited nonpitting thickened indurated plaques in the area of the upper back and posterior neck (Figure 1). There was redistribution of body fat with truncal obesity, thin extremities, and round facies. Raynaud's phenomenon, sclerodactyly, and nailfold capillary changes were not observed. The patient's CD4 cell count was 479/mL. His hemoglobin A1C level was 9% (4.3–5.8). He had a high serum glucose level of 209 mg/dL (70–110) and high total cholesterol at 253 mg/dL (128–219). Dexamethasone suppression test was normal. Antistreptolysin O titer was negative, and a paraprotein was not found by serum electrophoresis. 

**Figure 2:**
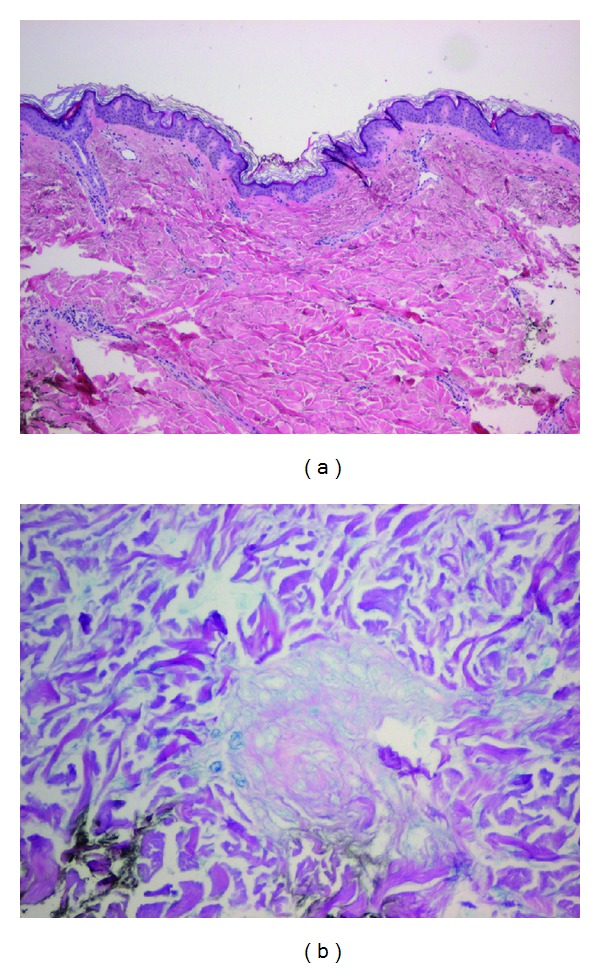
(a) Hematoxylin-eosin (HE) stain: thickened dermis with coarse collagen bundles. (b) Alcian blue dye: increased accumulation of mucopolysaccharides between large collagen bundles.

 Skin biopsy showed thickening of the dermis with coarse broad collagen bundles in a fenestrated pattern (Figure 2(a)). Deposition of acidic mucopolysaccharide between the collagen bundles was prominent and was confirmed by alcian blue staining (Figure 2(b)). The distinctive histological features were consistent with scleredema adultorum of Buschke. The patient was referred to an internist for more intense control of his diabetes.

## 3. Discussion

The cutaneous mucinoses are a heterogeneous group of disorders characterized by the accumulation of acid glycosaminoglycans (mucin) in the skin [3]. Scleredema adultorum of Buschke or scleredema is a form of cutaneous mucinoses with skin hardening of the posterior neck, shoulders, and trunk. The hands and feet are characteristically spared. In contrast to scleroderma or systemic sclerosis, scleredema is not associated with sclerodactyly, Raynaud's phenomenon, nailfold capillary changes, or serum autoantibodies. However, there have been rare cases of scleredema demonstrating extracutaneous involvement with mucopolysaccharide staining of the tongue, myocardium, and salivary glands [4]. 

Three variants of scleredema have been described. Type 1, identified by Buschke, is considered the classic type and follows an infection or febrile illness with resolution occurring in weeks to months. Type 2 tends to follow a slow, progressive course with an increased risk of developing paraproteinemia including multiple myeloma. Type 3 is termed scleroderma diabeticorum due to the association with diabetes mellitus [5]. Scleredema has a distinctive pathology. The dermis tends to be thickened, up to four times the normal way, with coarse swollen collagen bundles separated by wide spaces in which acid mucopolysaccharides are found [6].

No single form of treatment has been proven to be consistently beneficial. In most cases, glucose control has been the first step in treatment. Various immunosuppressants (IVIG, methotrexate, corticosteroids, and colchicine) have been tried without clear benefit. Physiotherapy is recommended in all cases. Ultraviolet A-1 phototherapy has been tried with good response [7]. 

In the last few years, cutaneous mucinoses have been reported with increased frequency in HIV patients. Among the cutaneous mucinoses, scleredema has been rarely seen in an HIV-infected patient, especially in AIDS-related insulin resistance and lipodystrophy syndrome.

After 1996, with the introduction of highly active antiretroviral therapy (HAART), there was a significant increase in the survival rate and improvement in the quality of life for HIV/AIDS patients. However, the prolongation of the lives of HIV-infected patients and/or the long-term use of novel, potent antiviral agents has generated new complications such as AIDS-related lipodystrophy syndrome [8]. 

Lipodystrophy is characterized by the redistribution of body fat from the limbs, face, and buttocks (lipoatrophy) to the central region of the body (lipohypertrophy) [8]. The excessive accumulation of abdominal fat and its ensuing insulin resistance will increase the risk of diabetes, dyslipidemia, and cardiovascular diseases. The long-standing diabetes, poor glucose control, and diabetic microangiopathy in these patients may contribute to the development of scleredema diabeticorum.
